# Is society caught up in a Death Spiral? Modeling societal demise and its reversal

**DOI:** 10.3389/fsoc.2024.1194597

**Published:** 2024-03-12

**Authors:** Michaéla C. Schippers, John P. A. Ioannidis, Matthias W. J. Luijks

**Affiliations:** ^1^Department of Organisation and Personnel Management, Rotterdam School of Management, Erasmus University Rotterdam, Rotterdam, Netherlands; ^2^Department of Medicine, Stanford University, Stanford, CA, United States; ^3^Department of Epidemiology and Population Health, Stanford University, Stanford, CA, United States; ^4^Department of Biomedical Data Science, Stanford University, Stanford, CA, United States; ^5^Department of Statistics, Stanford University, Stanford, CA, United States; ^6^Meta-Research Innovation Center at Stanford, Stanford University, Stanford, CA, United States; ^7^Department of History of Philosophy, Faculty of Philosophy, University of Groningen, Groningen, Netherlands

**Keywords:** Death Spiral Effect, complex adaptive systems, societal collapse, income inequalities, dysfunctional behavior, elite and masses, turnaround leadership, strengthening of democracy

## Abstract

Just like an army of ants caught in an ant mill, individuals, groups and even whole societies are sometimes caught up in a Death Spiral, a vicious cycle of self-reinforcing dysfunctional behavior characterized by continuous flawed decision making, myopic single-minded focus on one (set of) solution(s), denial, distrust, micromanagement, dogmatic thinking and learned helplessness. We propose the term *Death Spiral Effect* to describe this difficult-to-break downward spiral of societal decline. Specifically, in the current theory-building review we aim to: (a) more clearly define and describe the Death Spiral Effect; (b) model the downward spiral of societal decline as well as an upward spiral; (c) describe how and why individuals, groups and even society at large might be caught up in a Death Spiral; and (d) offer a positive way forward in terms of evidence-based solutions to escape the Death Spiral Effect. Management theory hints on the occurrence of this phenomenon and offers *turn-around leadership* as solution. On a societal level *strengthening of democracy* may be important. Prior research indicates that historically, two key factors trigger this type of societal decline: *rising inequalities* creating an upper layer of elites and a lower layer of masses; and *dwindling (access to) resources*. Historical key markers of societal decline are a steep increase in inequalities, government overreach, over-integration (interdependencies in networks) and a rapidly decreasing trust in institutions and resulting collapse of legitimacy. Important issues that we aim to shed light on are the behavioral underpinnings of decline, as well as the question if and how societal decline can be reversed. We explore the extension of these theories from the company/organization level to the society level, and make use of insights from both micro-, meso-, and macro-level theories (e.g., Complex Adaptive Systems and collapsology, the study of the risks of collapse of industrial civilization) to explain this process of societal demise. Our review furthermore draws on theories such as Social Safety Theory, Conservation of Resources Theory, and management theories that describe the decline and fall of groups, companies and societies, as well as offer ways to reverse this trend.

## 1 Introduction

Ants rely on each other for survival and often hunt for prey together. They use pheromones to locate each other and they follow the ones in front of them. This usually works quite well, although sometimes the ants get locked in what is called an “ant mill” or “Death Spiral.” This can happen when a subset of ants gets separated from the main foraging group and begin following each other. They start forming a continuously rotating circle, and the ants caught up in this Death Spiral often die from exhaustion. It has even been observed that dead ants are being pushed out of the circle, while the ants maintain their rounds. This “ant mill” or “circular milling paradox” seems to be the evolutionary price that army ants pay for an otherwise successful strategy of collective foraging (*cf.* Delsuc, 2003). The pathological, dysfunctional behavior is the other side of the coin of otherwise functional behavior. Rosabeth Moss Kanter, who spent years of studying declining organizations, concluded that a process similar to a Death Spiral may be happening to failing companies (Kanter, 2003). After years of success, these companies have trouble managing processes when the tide turns and problems occur. Instead of looking for solutions with an open mind, companies often get caught up in a Death Spiral, making decisions that seem rational, such as downsizing and centralized decision making (*cf.* Charan et al., 2002; Lamberg et al., 2017). Often these decisions worsen the situation instead of making it better, and self-destructive habits include denial, complacency and cost-inefficiency (Sheth, 2007). Sheth (2007) argues that denial of the new reality and internal turf wars, i.e., territorial impulse, are two dangerous self-destructive habits that can further send a company into decline. Companies are reluctant to admit they are in trouble and instead blame circumstances outside their control (Lorange and Nelson, 1985; Charan et al., 2002). Management research has also shown that long before the crisis within a company becomes apparent, the signs are there, but often go unnoticed or are ignored (Lorange and Nelson, 1985; Fitzgerald, 2005). Having to address these problems down the line, often leads to taking drastic steps and overreaction that may further fuel decline (Lorange and Nelson, 1985; Hafsi and Baba, 2022).

Using the metaphor of a corporate heart attack, Fitzgerald discerns a hidden, subtle and overt phase of decline (Fitzgerald, 2005). In the hidden phase, denial or willful blindness often prohibits management from taking (the right) actions. Against their better judgment, they hope if they ignore it, the market will not notice. In that phase, on average a third of a company’s competitive value is lost. If a new market challenge presents itself, the company is often unable to face the challenge. In the subtle phase, the decline becomes more obvious for those who are observant and know where and how to look and how to interpret what they see. By the end of this phase, often a full two-thirds of the company’s competitive value is lost. Unfortunately, many companies only start to admit and address the problem in the overt phase. By that time, the problems are so big and ingrained, that addressing them has become extremely difficult. While many managers do watch the company’s financials, they often fail to address other metrics such as market-share trends, customer turnover and staff satisfaction. Often these drivers are the earliest predictors of corporate performance. Important blockers of performance are distrust, bureaucracy and low performance expectations, while drivers are decisiveness, accountability and acknowledgement of work. It is key to identify and quantify early warning signals, e.g., an excess of staff, especially managers, a decrease in lower-level workers, tolerance of incompetence, and lack of clear goals (Lorange and Nelson, 1985). Reversing organizational decline starts with the realization and recognition that the organization is in decline. These danger signals should then be aligned with a concrete plan to change. A dialog between top-down and bottom-up is needed (Lorange and Nelson, 1985). If the company is able to take those steps, follow-up monitoring is needed to make sure the changes that are proposed and made are effective (Lorange and Nelson, 1985). While in the early phases underreaction may be the problem, in later phases, the danger comes from overreaction (*cf.* Lai and Sudarsanam, 1997; Hafsi and Baba, 2022).

We believe that similar processes may happen at the societal level. Recent examples of societal systemic shocks are 9/11, the 2008 global financial crisis and the COVID-19 crisis (Centeno et al., 2022). On a societal level, researchers studying policy success and failure have started to investigate the role of policy under- and over-reactions (Maor, 2012, 2020). Policy overreactions are “policies that impose objective and/or perceived social costs without producing offsetting objective and/or perceived benefits.” (Maor, 2012; p. 235). For instance, preemptive overreaction is a form of policy that will often rely on persuasion by presenting “facts” in a certain way, manufacturing a perceived threat, and using messages to swing the public mood (Maor, 2012). An example is the cull of all pigs in Egypt during the swine flu crisis in 2009, even though zero cases had been reported (Maor, 2012). An important explanation is that in such cases groupthink may play a role. Groupthink, the forced conformity to group values and ethics, has symptoms such as collective rationalization, belief in inherent morality, stereotyped views of outgroups, pressure on dissenters, and self-appointed mind guards (Janis, 1972, 1982a,b; Janis et al., 1978). Preemptive overreaction shows that one is taking forceful and decisive action against a perceived threat, that may never materialize, and motives could be political and/or monetary gain (Maor, 2012).

While the period before the COVID-19 crisis may have been characterized by relative policy underreaction to complex social problems, also referred to as “wicked problems,” such as hunger and poverty (Head, 2018; Head B.W. 2022), the current times may be characterized by overreaction to certain problems. The COVID-19 crisis seemed to be characterized by groupthink and escalation of commitment to one course of action, at the expense of other possible solutions (Joffe, 2021; Schippers and Rus, 2021). Initial low-quality decision-making was followed by decisions that made things worse (Joffe, 2021; Schippers and Rus, 2021). The sheer scale and severe disruption caused by these policies has increased inequalities (Schippers, 2020; Schippers et al., 2022), an important marker of societal decline (Motesharrei et al., 2014).

A meta-theory explaining such disruptive events is Complex Adaptive Systems Theory, a theory that suggests that developments in systems of many constituents are often non-linear and systems, such as societies, show unexpected and self-organizing behavior (Lansing, 2003; Holovatch et al., 2017). Moreover, “mechanisms like tipping points, feedback loops, contagions, cascades, synchronous failures, and cycles that can be responsible for systemic collapse are fundamental characteristics of any complex adaptive system, and can therefore serve as a useful common denominator from which to examine collapses” (Centeno et al., 2022; p. 71). In today’s society, our continued survival increasingly depends on tightly coupled complex and fragile systems (e.g., supply chains), over which no one has responsibility (Centeno et al., 2022). The potential for contemporary collapse makes it more compelling than ever to learn from past collapse for insight (Centeno et al., 2022) and to study global patterns of behavior. The normative basis for our paper relates to ‘the greater good’: What is deemed good for the thriving of humanity is seen as ‘good’ or ‘functional’ while what is seen as ‘bad’ or ‘dysfunctional’ is behavior or decisions that harm the thriving of humanity.

## 2 Downward spiral

In the current narrative and theory-building review we coin the term *Death Spiral Effect* to describe this type of overreaction and the resulting cascading effects in policies affecting the general public. We review the current literature and extend complex adaptive systems theory to construct our theoretical model of collapse and reversal of societal decline. We view the Death Spiral Effect as a specific form of complex (mal)adaptive behavior that accelerates decline and makes it hard to reverse decline. Making use of the ant mill metaphor, we theorize that a Death Spiral Effect emerges where a society gets caught up in a dysfunctional behavioral mode. Making use of this metaphor, we aim to aid theory building around this construct (Shepherd and Suddaby, 2016). We describe the elements of this vicious downward cycle, such as rising inequalities, dysfunctional behavior of both elite and masses, and rise of authoritarianism. We examine how the behavioral underpinnings of the resulting environment can lead to escalation through war, famine, and pandemics. While there is a rich literature on early warning signs and markers of societal decline, the underlying mechanisms have received much less attention and explanations often miss the depth that the psychological, sociological and management theories may offer. We draw on theories such as collapsology (the transdisciplinary study of industrial civilization risk of collapse), Complex Adaptive Systems Theory, Social Safety Theory (that focuses on friendly social bonds development and maintenance), Conservation of Resources Theory (that focuses on obtaining and maintaining resources), and general management theories that describe the decline of groups. We also use Social Dominance Theory to explain how and why the resulting inequalities are hard to reverse (Pratto, 1999; Pratto et al., 2006). Our work is also related to X-risk studies, a field of study that looks at existential risks for humanity (Torres, 2018; Moynihan, 2020): We recognize the importance of cascading risks, and hint at how catastrophic (X)-risks could potentially combine to jeopardize human survival (Torres, 2018; Baum et al., 2019; Moynihan, 2020; Undheim and Zimmer, 2023). We use a ‘Big Picture’ approach to these problems (Campbell et al., 2023). We then depict a possible upward spiral, dissecting what elements are needed to reverse the Death Spiral and build a society where people can thrive and prosper. In doing so, we contribute to theory building around the psychological and sociological drivers of societal decline (Swedberg, 2016). Our aim is to contribute to knowledge about societal decline and flourishing in order to enhance mankind’s chances of flourishing.

### 2.1 Crisis and crisis handling

Several authors have noted that societal decline has similar phases to organizational decline in companies, including early warning signs (Tainter, 1989; Downey et al., 2016; Scheffer, 2016; Jones, 2021; Demarest and Victor, 2022). Compared to decline in organizations, however, the scale at which this happens is bigger, the social consequences are more complex, and the decline may often be a more long-term process. The average lifespan of a company in the Standard and Poor’s 500 index in 2020 was 21.4 years (Clark et al., 2021) while some historical empires have lasted many decades or centuries (Taagepera, 1979). Another difference between organizations and society is that the outcome of decline can often not be buffered by society, such as would be the case in company decline. Also the hard outcomes (which may include war, famine and widespread disease) can be extremely hard to reverse (Downey et al., 2016). These three, war, famine and pandemics, we call the “Triangle of Death,” an expression coined by former Green Beret and combat correspondent Michael Yon (Yon and Peterson, 2022). However, Demarest and Victor (2022), p. 788 note that: “Even today the greatest challenge to knowledge coming from collapse studies–relevant not just for policy-makers and managers, but for the citizens of the entire society–is that no one really deeply believes that total collapse is possible”.

The process of societal decline is complex and may include social-ecological traps, or a mismatch between the responses of people and the social and ecological conditions they face, e.g., depletion of natural resources (Boonstra and De Boer, 2013; Boonstra et al., 2016). For the current review, we feel that the handling of the COVID-19 crisis may have been an example of overreaction making use of non-pharmaceutical interventions that accelerated existing societal problems, such as inequalities (Schippers, 2020; Schippers et al., 2022). Most countries opted for very similar solutions, with forced lockdowns and aggressive restrictions. Countries that chose a different course of action were highly criticized (Tegnell, 2021). Many countries eventually faced excess mortality rates that were highly unequal across groups, exacerbating preexisting inequalities (Alsan et al., 2021; Schippers et al., 2022). Over-reaction was fueled by (unreliable) metrics (Schippers and Rus, 2021; Ioannidis et al., 2022) and groupthink, resulting in irrational or dysfunctional decision making (Joffe, 2021; Hafsi and Baba, 2022). Furthermore, emotions during crises tend to run high, escalating the risk of harmful overreaction both by policy makers and the general public (Sunstein and Zeckhauser, 2010). Governments may suffer from an action bias, a tendency to take action whether it is needed or not, including excessive actions (Patt and Zeckhauser, 2000) despite information that the policies may do more harm than good (for reviews see Joffe and Redman, 2021, Schippers and Rus, 2021; Schippers et al., 2022). Unnecessary crisis response as a form of policy overreaction may sometimes occur as a way to shape voters perceptions of a decisive and active government (Maor, 2020). Excessive action and exercise of control over societal structures, e.g., public health, may enhance centralization of power and decision-making, and authoritarianism (Berberoglu, 2020; Desmet, 2022; Schippers et al., 2022; Simandan et al., 2023). When governments make use of mass media to spread negative information, a self-reinforcing cycle of nocebo effects, “mass hysteria” and policy errors can ensue (Bagus et al., 2021). This effect is exacerbated when the information comes from authoritative sources, the media are politicized, social networks make the information omnipresent (Bagus et al., 2021), and dissenting voices are silenced (Schippers et al., 2022; Shir-Raz et al., 2022). This may lead to a vicious cycle of ineffective dealing with crises, low-quality decision-making and dysfunctional behavior, intensifying the current crises and leading to new ones, and eventually societal decline and even collapse.

According to Holden (2005), p. 651, a complex adaptive system is “a collection of individual agents with freedom to act in ways that are not always totally predictable and whose actions are interconnected. Examples include a colony of termites, the financial market, and a surgical team”. An important element is emergence, the idea that complex global patterns can emerge from local interactions (Lansing, 2003). In the current paper, following authors such as Buckley (1998) and Lansing (2003), we view society as a complex adaptive system, with nested systems such as organizations and governments as part of the larger system. We suggest that the adaptive system is entering an unstable time, in the form of a series of crises. We extend Complex Systems Theory, by adding the Death Spiral as an element, where the system becomes so unstable, that actors within the network start to hold on to repetitive maladaptive (decision-making) behavior (Schwarzer, 2022); making the same decision over and over again, leading to unwanted results such as war, famine and pandemics (Triangle of Death) and ultimately the demise of society. In doing so, we explain the underlying mechanisms of societal decline and outline possibilities for reversal.

**Figure 1:**
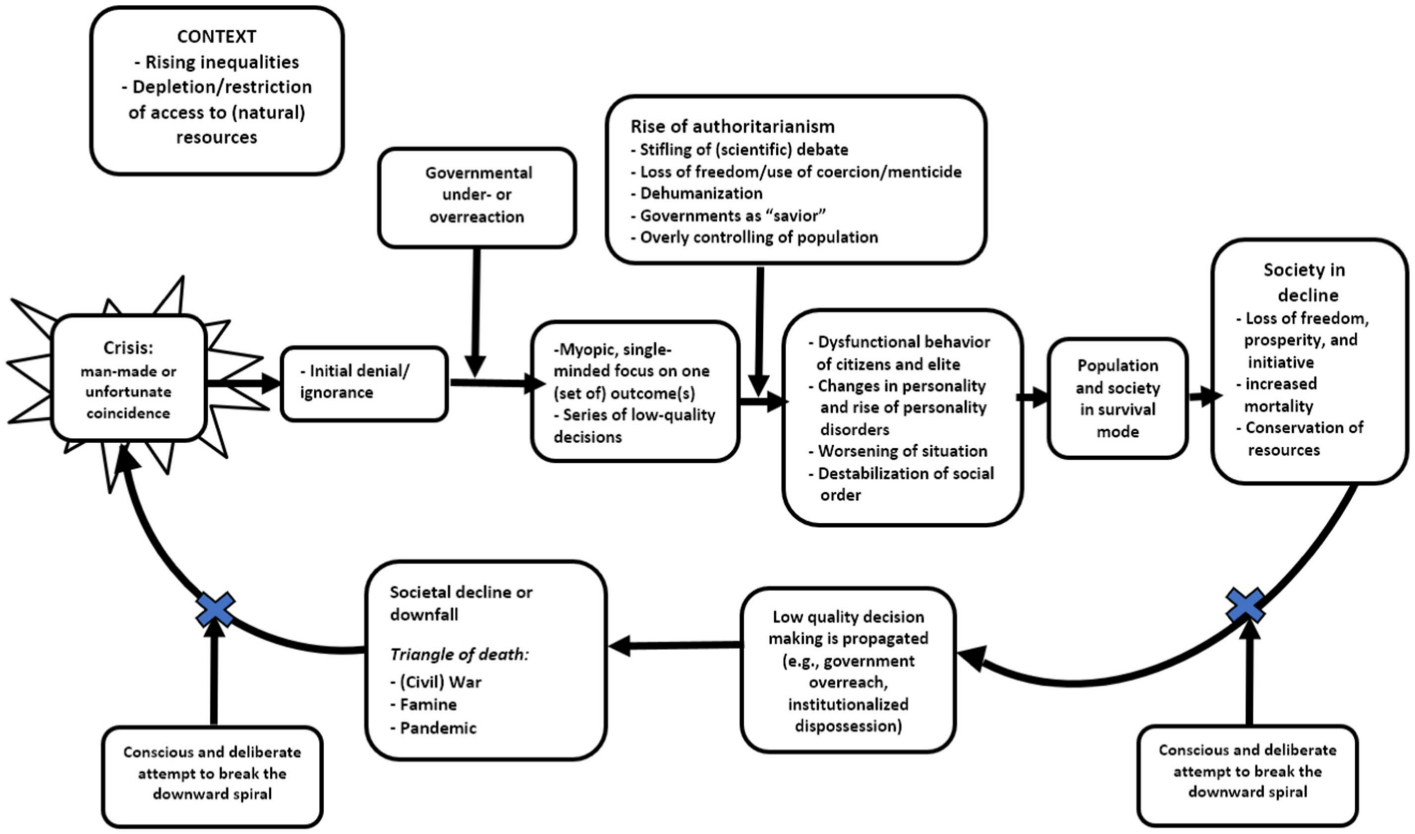
Death Spiral Effect: downward spiral of societies and/or groups in decline.

**Figure 2:**
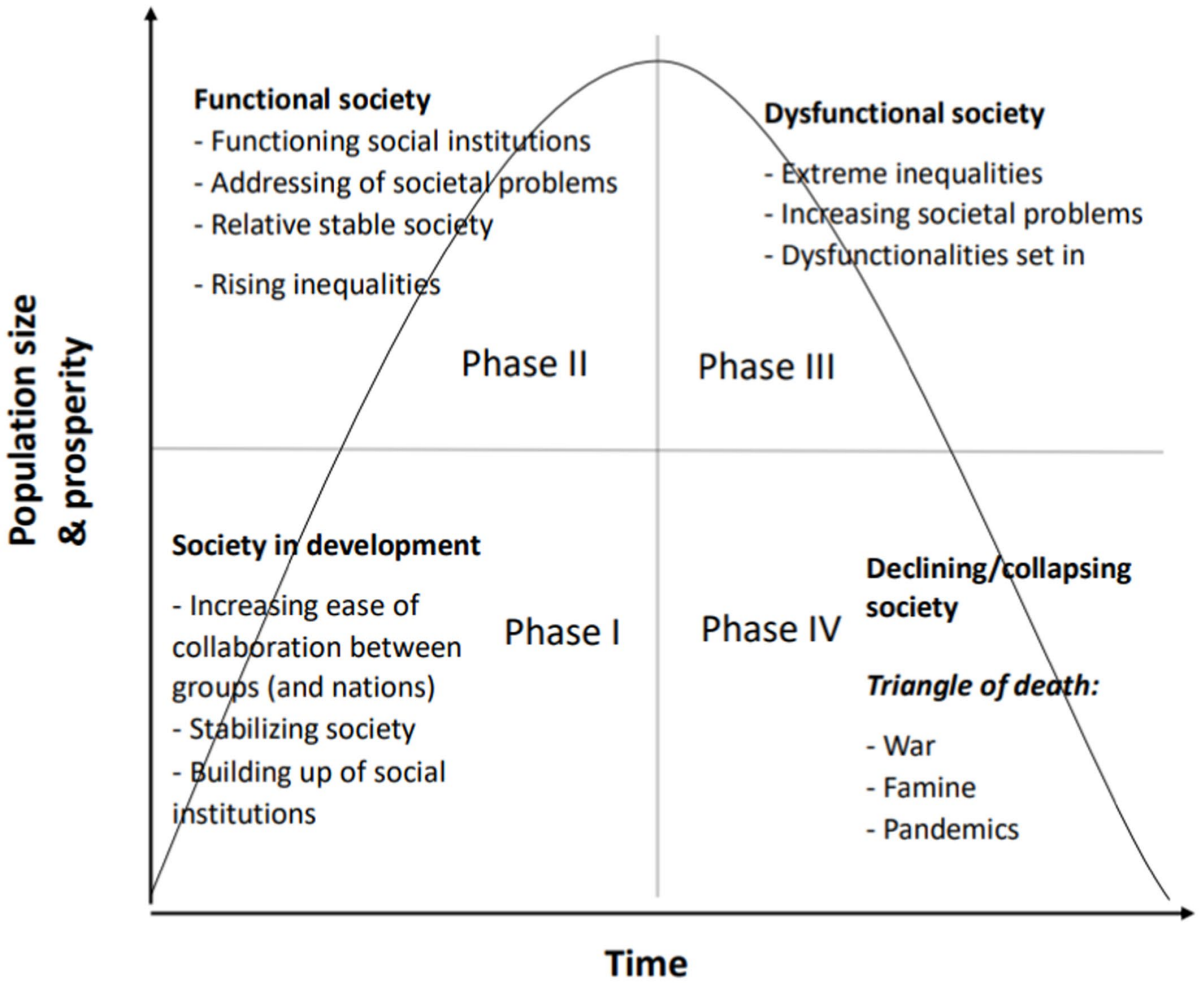
Death Spiral model of societies in decline.

Below we will first define and describe the process of a Death Spiral, and the similarities and differences between a Death Spiral and other concepts such as group think and mass formation. We will do this in the context of the more meta-theory of complex adaptive systems. Second, we will describe the elements of a societal downward (death) spiral, e.g., low-quality decision-making, rise of authoritarianism, and dysfunctional behavior of both the elite and masses (see Figures 1, 2). We will do so in the context of historical as well as current examples. Third, we describe the possibilities for an upward spiral, e.g., presence of a high-quality turn-around leadership, restoration of trust, and development of turnaround strategy.

### 2.2 Death Spiral considerations

When people and groups encounter difficulties or trauma (or sometimes for no apparent reason), they can start to make decisions that do not ensure survival, but seem self-destructive at best (*cf*. Balcombe and De Leo, 2022). They may make decisions to cope with the situations, but these can be characterized as mal-adaptive, non-adaptive, or semi-adaptive (Marien, 2009). Attempts to escape a downward spiral sometimes make it worse, by using counterproductive coping mechanisms (e.g., Freyhofer et al., 2021). The dysfunctional behavior continues if the spiral is not broken, and decline may follow from increasingly fragmented political institutions (*cf.* Kreml, 1994). In terms of Complex Adaptive Systems Theory, the system is then entering an unstable time, in this case a crisis (Centeno et al., 2022). When the system gets a blow, for instance from financial decline, depletion of resources, or other turns of fortune (Motesharrei et al., 2014), groups or societies may feel compelled to take action without considering carefully whether their decision-making process is valid (*cf.* Schippers et al., 2014). The threat-rigidity effect predicts a restriction in information processing and constriction of control under conditions of threat (Staw et al., 1981). The whole system becomes unstable and dysfunctional behavior sets in (Mohrman and Mohrman, 1983). The environment becomes generally stressful and threatening, eliciting more and more self-protective and rigid behaviors, that further threatens stability and group survival (Staw et al., 1981).

Finally, individuals and groups may tend to go around their lives in “circles” repeating the same mistakes, seemingly trapped in one behavioral mode. In organizations, similar Death Spiral pathologies can set in when changes in the environment do not invoke adaptation, but secrecy, blame, avoidance as well as passivity and learned helplessness (Kanter, 2003). In the general management literature, dysfunctional behavior is often described as a form of antisocial behavior, intended to bring harm (e.g., Giacalone and Greenberg, 1997; Van Fleet and Griffin, 2006). In the current paper, dysfunctional behavior is seen as counterproductive or ineffective behavior, that may have outlived its usefulness, and does not have the intended effect and may even have (unintended) harmful outcomes (Robinson, 2008). In companies, dysfunctional or counterproductive work behavior undermines efficiency and can range from social loafing (putting less effort when working as part of a group than when working alone), conflict and withdrawal to theft, fraud, bullying and even murder (Robinson, 2008). The more “civilized” forms of dysfunctional behavior, such as social loafing and withdrawal, are most prevalent (Robinson, 2008), and these can become much more common in organizations and societies that are in a downward spiral, and undermine individual autonomy. People feeling powerless in organizations exercising excess power are often triggered to perform counterproductive work behaviors (Lawrence and Robinson, 2007). During the COVID-19 crisis, withdrawal effects became more widespread and the crisis sparked changes in attitudes toward work as well as changing work behaviors inside organizations (Newman et al., 2022). For many workers, stress levels increased, and work performance declined (e.g., Vaziri et al., 2020; Kumar et al., 2021).

At the organizational level, decline often sparks dislike and distrust among managers, who then start to avoid one another, hide information and deflect blame (Kanter, 2003). People within the organization do not act in concert anymore and the dwindling success rate of their actions make them feel helpless (Kanter, 2003). In such situations, managers often resort to micromanagement: trying to control the actions of workers at a frustrating level of detail to steer them back to productivity. The pushback from workers will be to misbehave as a form of organizational resistance (Lawrence and Robinson, 2007), self-reinforcing cycles of micromanagement and counterproductive work behaviors (*cf.* Jensen and Raver, 2012; Cannon, 2022). A toxic work or societal culture may emerge and persist for some time, with fear as an overriding principle (Cannon, 2022). In society at large, the dangers of a “toxic discourse” around pending disasters (Buell, 1998; Horfrichter, 2000) may have paved the way for drastic measures taken to avoid such disasters (Schippers, 2020). However, some measures taken to prevent these hypothetical or expected future disasters have caused damage, leading to a steep increase in poverty and inequalities (Schippers et al., 2022). Besides many layoffs, many people reflected on their working life and subsequently decided to quit their job. The resulting “Great Resignation” seemed to be a world-wide phenomenon (Jiskrova, 2022; Sull et al., 2022; Del Rio Chanona et al., 2023). In the United States, monthly resignation rates were higher than in the previous 20 years (Statistics, 2021; Jiskrova, 2022). Note that many workers changed jobs and did not withdraw from the work force altogether (“Great Reshuffle”; Sull et al., 2022). However, at the beginning of 2021, more than 40% of workers were thinking of quitting, and a toxic work culture was mentioned as an important reason (Sull et al., 2022). At the same time decline in organizations was often triggered by the COVID-19 crisis and non-pharmaceutical interventions implemented to reduce viral spread, such as closing of restaurants and “non -essential” shops (Brodeur et al., 2021). As early as April 2020 in the United States, the number of active business owners decreased by 22% within just 3 months (Fairlie, 2020; Brodeur et al., 2021). Taken together with other effects such as rising inequalities, increase in immigration, changed labor market, damaged mental health and well-being, this is arguably a big shock to societal cohesion (Silveira et al., 2022), increasing state fragility and decreasing state legitimacy (Seyoum, 2020).

In both society at large, as well as in many companies, toxic cultures can ensue during crises (*cf.* Meidav, 2021). In such cultures, behavior that management or governments would like to see is rewarded, while many (mal)practices go unchecked, leaving room for fraud and corruption (*cf.* Kerr, 1975; Meidav, 2021; Breevaart et al., 2022). Indicative of such a toxic culture are: (lowered) level of helpfulness of people, (in)formality and (blind) enforcement of rules, underground avoidance of rules, feeling that things could be better but also feeling unable to change them, moaning “around the water cooler,” loss of morale, lack of initiative, top-down decision making, “double speak,” and lack of cohesion (Cannon, 2022). People are generally willing to do the right thing but find many roadblocks when they try (Myers, 2008). Moreover, historical research has shown that people fall back on “overlearned” comfort behavior, and biases become entrained again. For instance, a fallback on preference for ingroups ensures that during crises diversity efforts in companies are reduced and inequalities rise (Meidav, 2021). During organizational change, employee misconduct increases (Ethics and Compliance Initiative, 2020; Meidav, 2021) including even antisocial behavior (Belschak et al., 2018).

### 2.3 Death Spiral Effect: definition and key characteristics

Based on the above considerations, here we formally define the Death Spiral Effect as: *A vicious cycle of self-reinforcing dysfunctional behavior, characterized by continuous flawed decision making, myopic single-minded focus on one (set of) solution(s), resource loss, denial, distrust, micromanagement, dogmatic thinking and learned helplessness.* The Death Spiral is often initiated by an external or internal event (e.g., crisis) causing a trauma or emotional response. In the case of man-made crises, a positive feedback loop of perverse incentives may cause a stable society to spiral into disorder (Centeno et al., 2022). The Death Spiral Effect sets in when a cascade of events is difficult to stop once set in motion (*cf.* Centeno et al., 2022). On a societal level this spiral results in increasing gap between elite and masses, rising authoritarianism and massive resource loss. For instance, it has been noted that ancient civilizations on the brink of collapse used scarce resource for megalomaniac projects, such as huge temples, in a desperate attempt to legitimize declining institutions, but ultimately staying on the course toward disintegration (Demarest and Victor, 2022).

A Death Spiral is characterized by: (1) initial denial of the problem; (2) continuously and repeated flawed decision-making, often trying to fix the problem with the same ineffective solution over and over again; (3) increasing secrecy and denial, blame and scorn, avoidance and turf-protection, passivity and helplessness; (4) worsening of the situation, and a continuous (series of) crises following, further triggering a “survival mode” and tunnel vision, and (5) the felt or observed inability to escape or snap out of the ineffective cycle of decision-making. Other characteristics that emerge when the Death Spiral becomes apparent are: (1) a negative and distrustful atmosphere; (2) micromanagement: individuals, management or government trying to increase the number of (strict) rules and a focus on the adherence to those rules at the expense of effective problem-solving; and (3) censorship of opinions and knowledge outside the official narrative. These elements may be present to variable degrees concurrently and may reinforce each other. As the downward cycle continues, and resources loss escalates, the *desperation principle* may set in: a defensive mode in which people or groups aggressively and often irrationally try to hold on to the little resources that are left (Hobfoll et al., 2018), instead of thinking on how to snap out of the situation altogether.

In Figure 2, we discern four phases of societal development and demise: In phase I society is developing and growing. During this phase groups are working together without large problems. Social intuitions are founded and strengthened. In phase II, a functional society is relatively stable although, social inequalities are already increasing in this phase. In phase III, a dysfunctional society, the seeds of discontent sown during phase II have now matured: social inequalities are becoming more extreme, resulting in an increasing number of societal problems and an uptake in societal dysfunctionalities. Governments often react via centralization of power, and a rise in authoritarianism instead of involving the general public in solving societal problems. In phase IV, we see a declining or collapsing society. If the problems of decline, that started in Phase II and III, are not addressed then society will decline and may eventually collapse. Collapse is characterized by the Triangle of Death: war, famine and pandemics (Karabushenko et al., 2021; *cf.* Kuecker, 2007; Yon and Peterson, 2022; see also Figure 1).

### 2.4 Differences from other concepts

While we define the Death Spiral Effect as a specific form of collapse within an adaptive complex system, the concept of a Death Spiral is an umbrella concept that has some overlap with but also distinct features from some other concepts, such as group think, mass formation, Abilene paradox, and group polarization. In Table 1 we list those concepts and give an overview of similarities and differences versus the Death Spiral Effect. All those concepts deal with forms of dysfunctional decision-making. However, the main difference is a combination of the repetitiveness of the dysfunctional decision-making process, and the stubborn and prolonged effect of the subsequent series of decision-making (See Table 1).

**Table 1:** Death Spiral Effect compared to other related concepts.

Concepts → Attributes ↓	Death Spiral Effect	Mass formation	Groupthink	Abilene paradox	Group polarization
Other names for the concepts	Ant mill effect	Crowd formation, Group formation (Hernández, 1988)	None	None	None
Concise definition	A process where individuals, groups and/or societies get stuck in a behavioral mode that leads to repeated subpar decision making, which may result in the collapse of a society.	The mass behaves like a swarm or a group of molecules, because people are in an altered psychological state (Desmet, 2022; p. 93, Schippers et al., 2022). The end result is that the masses adapt to a totalitarian mindset, where deviation of the main narrative is not accepted.	“Mode of thinking in which individual members of small cohesive groups tend to accept a viewpoint or conclusion that represents a perceived group consensus, whether or not the group members believe it to be valid, correct, or optimal. Groupthink reduces the efficiency of collective problem solving within such groups”. (Schmidt, 2016).	“Organizations frequently take actions in contradiction to what they really want to do and therefore defeat the very purposes they are trying to achieve”. (Harvey, 1974; p. 66). The Abilene paradox describes a self-defeating process.	The tendency of a group to make decisions that are more extreme than the initial inclination of its members. These more extreme decisions tend to favor greater risk if people’s initial tendencies are risky, and caution if people’s initial tendencies are cautious.
First publication on the concept	On the Death Spiral Effect in actuarial science and health economics: “Adverse Selection in Health Insurance” (1998) by David M. Cutler (1965-present) and Richard J. Zeckhauser (1940-present) (Cutler and Zeckhauser, 1998). On the ant mill effect in animal behavior *Edge of the Jungle:* 291–294 (1921) by Charles William Beebe (1877–1962) (Beebe, 1921). N.B. in this paper we develop the Death Spiral Effect further and apply it to society as whole.	In English: Hannah Arendt, *The origins of Totalitarianism* (2017) [1951] (Arendt, 2017). In German: Massenbildung in *Massenpsychologie und Ich-Analyze* (1921) by Sigmund Freud (1859–1939) (Freud, 1921). In French: *La Psychologie des foules* (1895) by Gustav Le Bon (1841–1931) (Le Bon, 1895).	For the popular audience:. Groupthink’ (1952) by William H. Whyte Jr. (1917–1999) (Whyte, 2012). In scholarship: by Irving Lester Janis (1918–1990) (Janis, 1983).	“The Abilene paradox: The management of agreement” (1974) by Jerry B. Harvey (1935–2015) (Harvey, 1974).	James A. F. Stoner (1935-present) in an unpublished master thesis as ‘risky shift’ (Stoner, 1961).
Stuck in a behavioral mode	Yes and thereby ensuring suboptimal decisions.	To some extent, behaving like a swarm.	No, but stuck in a mental framework.	People engage in behavior none of them wants to engage in, but they do not address the issue.	Conformity seems to contribute to the behavior.
Unit of analysis	Individual, group, society.	Society or the mass(es) (Arendt, 2017; p. 403)	Group	Group	Group
Level on which the concept operates	Society, but the role of groups and individuals are also described.	Society and groups (if the society is too small in population: mass formation cannot take effect; Arendt, 2017; p. 403–406).	Groups	Groups	Groups
Viewing society as a swarm	Yes	Yes (Desmet, 2022, Schippers et al., 2022; p. 4)	No	No	No
View of the group	As an entity, but also consisting of individuals and groups that can make their own decisions and “break” away from the ant mill.	The concept applies to societies as a whole and groups. The group behaves as a swarm (Desmet, 2022, Schippers et al., 2022; p. 4) or “super individual” (Desmet, 2022; p. 125–126). Desmet borrows the concept of super individual to describe the crowd from Nikolaas Tinbergen (Tinbergen, 1946).	“Just a sum of fragmented individuals” (Kim, 2001).	“As a single organism” (Kim, 2001).	Social group behavior, sometimes as a network of individuals (e.g., Zhang et al., 2020).
Micromanagement	Is part of the concept.	Desmet (2022) describes: a. regulation mania’ (pp. 79–80).	No	No	No
Descriptive and/or explanatory	Descriptive and explanatory	Descriptive	Descriptive	Descriptive	Descriptive and explanatory
Individuals attitude toward the issue	Active	Active/passive	Active (Kim, 2001; p. 180–181, 187)	Passive (Kim, 2001; p. 180–181, 187)	Active
Self-censorship	Yes	Yes	Yes (Janis, 2003)	Yes	Unknown
The concept is concerned with decision making moments and processes	Yes	Yes	Yes	Yes	Yes
Responsibility for faulty decision making	Elites and in a later stage the masses	Elites are responsible and the crowd is complicit. The crowd and the leaders hypnotize each other.	Groups	Individuals	A shared responsibility
Effect on risk taking behaviors and/or decision making	Decision makers get stuck on an unproductive path.	Mass formation leads to decisions making based on wrong assumptions and power that cannot be challenged.	Groupthink leads to defective decision making.	Decisions that are made do not align with the interests/goals of the organization.	More likely to take risk.
Individuals’ perception of the decision at the time of the decision making	Not specified	The individual’s identity has been subsumed by the group identity (Desmet, 2022).	“Made of their own free will, and hence took an air of attachment for that decision”. (Kim, 2001; p. 185).	“Coerced into making a decision, and then took an air of detachment from that decision.” (Kim, 2001; p. 185).	Not specified
During group decision-making, individuals’ conditions could be assessed as:	Dysfunctional and sometimes even manipulated/ brainwashed in order to go as a group in one direction.	“The fanaticized members can be reached by neither experience nor argument, identification with the movement and total conformism seem to have destroyed the very capacity of experience, even if it was torture or the fear of death.” (Arendt, 2017; p.403).	“Preoccupied by group illusions such as invulnerability and unanimity → no dilemma” (Kim, 2001; p. 185).	“Firm commitment to their own views leads to the dilemma (expressing their views vs. going along with the misperceived collective reality)” (Kim, 2001; p. 185).	Crowd mentality where group decisions become more extreme than when acting alone.
Affective state of individuals	Depends on the situation	Fearful	“Group euphoria” (Kim, 2001; p. 185)	“Pain, incompetence, frustration, irritation or anger” (Kim, 2001; p. 185).	Mob mentality, group emotions propagate within the group (anger, euphoria, etc.).
Internal group status after decision making	Not specified	Not specified	“Esprit de corps or loyalty to the organization; higher cohesiveness” (Kim, 2001; p. 186).	“Conflict; lower or after crumbled cohesiveness” (Kim, 2001; p. 186).	Not specified
Most influential independent variable	Series of dysfunctional decisions that increases inequality gap between elite and masses.	Fanaticism (Arendt, 2017; p. 402–403) As long as individuals can stay members of the movement, they are prepared to sacrifice themselves.	“Fear of separation” (Kim, 2001; p. 186)	“Cohesiveness” (Kim, 2001; p.186)	Persuasive argumentation (Isenberg, 1986)
Energy state	Can be high and low energy.	Can be both high and low energy.	High energy (Kim, 2001; p.184, 188)	Low energy (Kim, 2001; p. 184, 188)	Does not apply.
Can be subsumed as part of Death Spiral	Not applicable	Can be subsumed.	Can be subsumed.	Not applicable	Can be subsumed by the Death Spiral Effect and groupthink.
Stereotyping of enemy groups as evil and/or targeted for elimination.	Not always	Yes	Yes (Janis, 2003)	Not applicable	Sometimes
The type of pressure exercised on members of the group/society	Normative and informational influence by elite.	Normative and informational influence.	Pressure “is directly applied to anyone who momentarily expresses doubts about the group’s shared illusions. Such pressure often is masked as amiability, in an attempt to. domesticate’ the dissent, so long as doubts are not expressed outside the ingroup, and fundamental assumptions are not challenged”. (Cooke, 1999; p. 113).	Not applicable	Normative and informational influence
Morality	Elite appeals to morality to steer behavior of masses.	Under the condition of mass formation, the crowd has “a strong tendency to surrender to impulses that, under normal circumstances, would be considered radically unethical”. (Desmet, 2022; p. 92).	Group members. believe unquestionable in the inherent morality of their ingroup’ and predisposing. members to ignore the ethical or moral consequences of their decisions’ (Janis, 2003; p.264).	Not applicable	Sometimes appeals to morality.
The illusion of invulnerability	Yes	Yes	Yes (Janis, 2003)	No	No
Unanimity	Yes	The individual disappears in the group which acts like a new. super individual’. (Desmet, 2022; p. 125–126).	‘An illusion of unanimity exists with the group, with silence assumed as concurrence with the majority view’. (Cooke, 1999: 113).	Yes	Yes
Mind guards	Are part of the concept	Are part of the concept	Are part of the concept (Janis, 1983; Cooke, 1999; p.113)	Not necessarily	Sometimes, not necessarily

The Death Spiral Effect differs from groupthink, group polarization and the Abilene paradox in that groupthink, group polarization and the Abilene paradox are often related to a more finite series of decisions around one topic or outcome (e.g., the invasion of the Pig Bay) and focuses more on group harmony and agreement (Janis, 1972, 1982a,b; Harvey, 1974). Thus, while groupthink, group polarization and the Abilene paradox may often be part of a Death Spiral, a Death Spiral is more long-lasting, pervading, and pathological dysfunctional behavior and affects many aspects of a person’s life, a team, a company or even the whole society. At a certain moment, similar to groupthink, self-appointed mind guards appear, but the scale is much bigger. The Death Spiral Effect takes groupthink a step further, it can lead to the collapse of a full society.

Mass formation has also been offered as an explanation for what is happening in society (Desmet, 2022; Schippers et al., 2022). This theory sees the people in society as a swarm, that will move in one direction, following a single narrative. The mass formation concept does not have a going around in circles’ element, that the Death Spiral has. The swarm-like element in this theory states that people do attend to others’ behavior and copy that behavior (Bak-Coleman et al., 2021; Desmet, 2022). Swarm-dynamics are also studied within complex adaptive systems research (Huepe et al., 2011). While mass formation can be part of the Death Spiral Effect, and irrational group behavior is an element of this effect, the Death Spiral gives a broader explanation of what happens if (groups of) people get stuck in this cycle.

The dysfunctional behavior shown in a Death Spiral also includes micromanagement, a leadership style that stifles creativity and innovation (Allcorn, 2022) and has been pointed out to be a danger in terms of human freedom and an open society (Esfeld, 2022; see Table 1). “Tit for tat” is a concept from game theory, that is somewhat similar to the Death Spiral Effect in that parties get stuck in a behavioral mode, reaching suboptimal results for the involved parties, while it would be possible for the parties to change their behavior (and thereby get better results). The key difference is that the scope of the Death Spiral Effect is much broader, with more far-reaching implications and ripple effects.

### 2.5 Societies in decline: Death Spirals throughout history

Scientists have offered a variety of explanations for the collapse of civilizations through ancient and modern history (Tainter, 1989; Spengler, 1991; Bunce et al., 2009; Scheffer, 2016). Oftentimes, a combination of factors may play a role in societal decline (Jones, 2021). Nevertheless, recurrent patterns operate (Jones, 2021). Oftentimes, markers of decline are clear, and the decline may have set in long before the collapse (Scheffer, 2016). The study of societal collapse, collapsology, is traditionally studied by historians, anthropologists and political scientists. Also, experts in cliodynamics and complex systems have joined this field, although experts within management and psychology to date could potentially have much to offer in terms of behavioral explanations. Similar to the initial phase of decline in companies, societies tend to act too late, they resist change until smooth adjustments have become impossible (Scheffer, 2016). The “sunk cost effect,” based on escalation of commitment prevents people from leaving and abandoning their property, ways of living and beliefs, even when the need to do so becomes apparent (Janssen et al., 2003; Scheffer, 2016). Also, oftentimes elites may have a vested interest in maintaining the status quo (*cf.* Pratto et al., 2006; Wilkinson and Pickett R. G., 2009).

From a psychological point of view, trauma causing a shift in behavioral mode from functional to dysfunctional seems key to understanding the Death Spiral Effect. From a biological point of view, collapse can be viewed as inevitable after a period of large population growth (Downey et al., 2016). As complex systems, common factors may contribute to decline, and these may have ripple or cascading effects (Diamond, 2013). For a long time, the Malthusian catastrophe (the idea that the population growth outgrows the (linear) food supply, causing mass starvation and deaths) was perceived as a major threat (e.g., Diamond, 2013; Ramya et al., 2020). However, within a complex agricultural system, it seems possible to feed a growing world population. Also there seems to be general agreement in the literature that food shortages in past times were not the sole cause of societal collapse, and maybe even more a consequence of societies inability to deal with their problems (Diamond, 2013). Erosion of established systems and resulting lack of loyalty to established political institutions plus an increase in inequalities are all markers of decline (Turchin, 2007; Diamond, 2013; Van Bavel and Scheffer, 2021). In the interconnected globalized ‘system-of-systems’, ‘a failure in one part could lead to disaster across the whole structure’ (Centeno et al., 2022; p. 61). Some see signs that society may be at the brink of collapse (Page, 2005), and that while the scale of disaster can be unprecedented, lessons from the past in terms of complex systems are still relevant today (Centeno et al., 2022). Poor institutional choices result in an inability to solve collective action problems (Page, 2005). It has recently been noted that we live in a great third power shift in modern history, after the first, the rise of the Western world since the 15th century, and the second, near the end of the 19th century, the rise of the United States (Peters et al., 2022). The current power shift is defined by a rise of China, India, Brazil and Russia. An important problem that the US are dealing with is not only the growth of economic inequalities, which are huge, but also political division of society, military overreach and financial crises (Peters et al., 2022). The power elite have positions that enable them to make decisions that have far-reaching consequences for ordinary men and women. They are also often in a position that they can influence politicians an pressure groups (Mills, 2018). At the same time, some authors refer to a netocracy, a global upper-class with a power-base derived from technological advantage and networking skills. The new underclass, or masses, is called consumtariat, whose main activity is consumption, regulated by those in power (Bard and Söderqvist, 2002). Generally, what becomes apparent in the literature is that rising inequalities, which represent basically a growing divide between elite and masses, are an important and potentially reversable marker of societal decline (Moghaddam, 2010; Diamond, 2019).

### 2.6 Repeated low-quality decision-making

In a society in decline, the rate of decline and possible reversal are codependent on the governmental responses (Toynbee, 1987; Hutton, 2014). In some cases, there will be inaction, if a threat is not perceived as needing urgent action, but equally devastating can be overreaction to a threat (Maor, 2012; Maor, 2020; Hafsi and Baba, 2022). An action bias, a bias favoring action over inaction, often occurs when incentives to take action are bigger than incentives to refrain from action (Patt and Zeckhauser, 2000). After a while of ignoring warning signs, a tendency to react too strongly may take over, and this may also include suboptimal decision-making (Lorange and Nelson, 1985). When a crisis is over, decision makers may often not carefully consider all pros and cons. Taking these kinds of actions is more common than taking preventative, anticipatory actions, such as health advice, action to prevent a health crisis, and actions to prevent an environmental crisis (Patt and Zeckhauser, 2000; Magness and Earle, 2021). In the COVID-19 and accompanying economic crisis for instance, there is evidence of such an action bias (Winsberg et al., 2020; Magness and Earle, 2021, p. 512; Schippers et al., 2022). People often assume that a big problem needs harsh and drastic solutions, while less drastic, but precise solutions, as well as targeted, evidence-based interventions can work better than aggressive solutions (*cf.* Brown and Detterman, 1987; Wilson, 2011; Walton, 2014). Action bias, along with escalation of commitment and sunk cost fallacy may have played a role in the suboptimal decision-making processes surrounding the COVID-19 crisis (Schippers and Rus, 2021). Combined with the (in hindsight) overestimation made by experts of the expected infection fatality and of the buffering effects of several aggressive measures (Chin et al., 2021; Ioannidis et al., 2022; Pezzullo et al., 2023) led to a disastrous chain of self-perpetuating decision-making (Magness and Earle, 2021; Murphy, 2021). Instead of dialing back, the general political climate and response doubled down on the measures and on defending a narrative in their support, leading to a Death Spiral of low-quality decision making and serious consequences.

### 2.7 Key marker of societal decline: rising inequalities

In current society, there are some clear signs of societal decline. While dwindling resources are not always apparent in declining societies, a key marker is hierarchical order and an elite with plenty of access to resources while the masses have increasing difficulties to survive (Diamond, 2013; Rushkoff, 2022). Recently, a rather steep increase in inequalities has been observed (for a review see Schippers et al., 2022). This increase is partly caused by wage inequality, which the last 40 years has sharply increased in developing countries (Acemoğlu and Restrepo, 2021). Wage inequality is for a large part caused by automation (Acemoğlu and Restrepo, 2021). While poverty decreased since the 19th century (Sullivan and Hickel, 2023), there are now clear signs that this trend is being reversed. Economic inequality has been found to have a range of effects such as reducing mental and physical health (Wilkinson and Pickett R., 2009; Pickett and Wilkinson, 2015), decreasing trust, cooperation and social cohesion in society (Gustavsson and Jordahl, 2008; Elgar and Aitken, 2010; Van de Werfhorst and Salverda, 2012), heightening violence and social unrest (D’Hombres et al., 2012; Jetten et al., 2021) and increasing support for autocratic leadership (Jetten et al., 2021). Rising inequalities may thus have more far-reaching consequences and destabilizing effects than commonly believed, also via the effect on citizens’ sociopolitical behaviors and decreased social cohesion (Van Bavel, 2016; Jetten et al., 2021). Since the global financial crisis of 2008, this trend toward rising inequalities has become more visible (Jetten et al., 2021). Health within a population gets progressively worse alongside a development of decreased economic equality. Societies with relative equal levels of income commonly also have low levels of stress and high levels of trust, and people in such societies are generally cooperative. In unequal societies distrust rises as the rich fear the poor, they worry to safeguard their wealth, while the poor suffer from stress, status anxiety and bitterness (Wilkinson and Pickett R., 2009; Wilkinson and Pickett R. G., 2009). Health and life expectancy lowers for the poor, unemployed and low-level employees (Smith et al., 1990; Marmot and Shipley, 1996; Neckerman and Torche, 2007; Boehm et al., 2011). Importantly, economic inequality has also been described as a downward spiraling effect of social problems. These include teenage pregnancies, with babies born to such mothers at greater risk of educational failure, juvenile crime and becoming teenage parents themselves, with decreasing health, and increasing imprisonment of those lowest on the social ladder (Wilkinson and Pickett R., 2009). On a grander scale, societies fall apart and societal dysfunction rises when an ever increasing group of have-nots are unable to sustain themselves, let alone earn the money and produce the food to sustain the rich, and the difference between the elite and masses have become too big to bridge (Wilkinson and Pickett R., 2009, Wilkinson and Pickett R. G., 2009).

Note that while most social problems are bigger in unequal countries, suicide and smoking levels are often higher in contemporary relatively equal societies, as aggression and violence is turned inward, and often will be directed at the self, as people tend to blame themselves when things are not great (Wilkinson and Pickett R., 2009). Inequality may be at the root of many problems in societies and more equal societies do better on almost all fronts (Marmot and Shipley, 1996; Wilkinson and Pickett R., 2009; Boehm et al., 2011).

### 2.8 Historical examples of rising inequalities and societal decline

Prior to the 19th century, most unskilled laborers were able to provide for a family of four (Sullivan and Hickel, 2023). A review on wages and mortality since the 16th century showed that in general extreme poverty was not widespread, with the exception of severe social disruption and dislocation, such as war, famine and institutionalized dispossession. Interestingly, the rise of capitalism initially caused a dramatic *decrease* of human welfare, in terms of a decline in wages below subsistence level. In several regions, such as Northwest Europe, progress in terms of human welfare only began in the 1880’s, and in other regions as late as the mid-20th century. This period was characterized by anti-colonial and social political movements, and a redistribution of incomes as well public provisioning systems and the welfare state (Sullivan and Hickel, 2023).

Going back even further, during the decline of the Roman Empire, a Death Spiral seems to have been apparent. Even when the end was near, instead of trying to address the problems, there was unrealistic and excessive optimism about the future, and adherence to the past (Grant, 1976). In the earlier periods of the empire, the elites were willing to offer lives and treasure in the service of the common interest, while in the period of decline, the elites became increasing selfish (Turchin, 2007). This went hand in hand with a decline in dearly held values such as thinking for the common good and virtues, enlarged bureaucracies and a rise in inequalities with steep increase in enrichment of the richest 1 percent in Rome, and an impoverishment of the middle classes (Goldsworthy, 2009).

*“(…) the richest 1 percent of the Romans during the early Republic was only 10 to 20 times as wealthy as an average Roman citizen. (…) By around A.D. 400, just before the collapse of the empire and when the degree of wealth inequality reached its maximum value, an average Roman noble of senatorial class had property valued in the neighborhood of 20,000 Roman pounds of gold. There was no “middle class” comparable to the small landholders of the third century B.C.; the huge majority of the population was made up of landless peasants working land that belonged to nobles. These peasants had hardly any property at all, but if we estimate it (very generously) at one tenth of a pound of gold, the wealth differential would be 200,000! Inequality grew both as a result of the rich getting richer (late imperial senators were 100 times wealthier than their Republican predecessors) and those of the middling wealth becoming poor, and indeed destitute.”* (Turchin, 2007; pp. 160–161).

This rise of inequalities seems an overarching theme in many collapsing empire analyses (Turchin, 2007). The work of Turchin describes a series of nested cycles of periods of relative prosperity and plenty, leading to an increase of population, but also to growing inequalities and dysfunctionality. Inequality affects asabiyya,^1^ or social cohesion, defined by Turchin as: “the capacity of a social group for concerted collective action” (Turchin, 2007; p. 6). Asabiyya is generally high in times that empires are rising and low when empires are in decline (Turchin, 2007). Similar to the “Universe 25” experiment (described in par. 1.1.10), this in turn leads to a breakdown in collaborative efforts and precedes a period of scarcity. In the next phase, disease, hunger, violence and war then lead to a rapid decline and often collapse of civilization (Turchin, 2007; see Figure 2).

### 2.9 COVID-19 crisis and rising inequalities

In the context of the COVID-19 crisis, some have stated that this is a great leveler and that “we are all in this together,” however, this is clearly not the case: vulnerable groups have been unevenly negatively impacted (Ali et al., 2020). Inequalities have risen steeply since 2020 (Schippers et al., 2022). While this trend was already visible before the pandemic started (for a review see Neckerman and Torche, 2007), especially billionaire wealth increased dramatically early during the crisis (Schippers et al., 2022; Inequality, 2023). Between March 18, 2020, and October 15, 2021, billionaires’ total wealth increased over 70%, from 2.947 trillion to 5.019 trillion, and the richest five saw an increase in 123 percent. Since then, gains have decreased modestly, because of market losses (Collins, 2022). Corporate profits also spiked as giant corporations used the excuse of crisis-related supply chain bottlenecks to drive up the prices of gasoline, food, and other essentials (Inequality, 2023). While CEO pay increased, general worker pay lagged behind, increasing the CEO-worker pay gap in the United States (Inequality, 2023). To prove this in 2019 average CEO pay was $12,074,288 *per annum* compared to a median worker yearly pay at the 100 largest low wage employers of $30,416 in the United States; in 2020 yearly average CEO pay was 13,936,558 (a 15.42% increase) for workers it was 30,474 (a meager 0.19% increase; Inequality, 2023).

In effect, global billionaires made 3.9 trillion dollars by the end of 2020, while global workers earnings fell by 3.7 trillion, as millions lost their jobs around the world (International Labour Organization, 2020; Berkhout et al., 2021; p. 12). The lowest-income workers were hit the hardest. In total, it has been estimated that during the crisis, by 2021, 150 million people were driven into extreme poverty (Howton, 2020). With widespread continuing demise, even the rich may start to lose. The crisis has worsened many other aspects of inequality, such as educational, racial, gender, and health inequalities (Byttebier, 2022; for a review see Schippers et al., 2022). Nevertheless, the elite may continue to centralize power and make decisions that are not in the interest of most people (Desmet, 2022). As the “masses” end up being in a downward spiral of dwindling incomes, not being able to pay for essentials, such as food, gas, and medicine, they may experience significant financial barriers and may avoid health care in order to save on costs (Weinick et al., 2005), leading to worsening health status for millions (Schippers et al., 2022). Socio-economic determinants of health are often the result of persistent structural and socio-economical inequalities, exacerbated by the COVID-19 crisis (Ali et al., 2020; Schippers, 2020). The term *syndemic* describes “a set of closely related and mutually reinforcing health problems that significantly affect the overall health status of a population, against the background of a perpetual pattern of deleterious socio-economic conditions” (Bambra et al., 2020; Byttebier, 2022, p. 1036). Prior pandemic crises such as the Spanish flu and other economic shocks led to an increase in inequalities and unequal health and wealth outcomes (Bambra et al., 2020). Sudden economic shocks, such as the collapse of communism, are related to an increase in morbidity, mental health decline, suicide, increased ill health and deaths from substance use (Bambra et al., 2020). These effects were experienced unequally in poorer regions, and among low-skilled workers, exacerbating health inequalities (Bambra et al., 2020). Interestingly, after the 2008 financial crisis, countries that chose not to cut back on health and social protection budgets, had better outcomes than countries that made austere cuts in those budgets (Stuckler and Basu, 2013; Bambra et al., 2020). In current times, people lower on the social ladder bore the brunt of the negative side effects of the measures, in health, lifestyle changes as well as decrease in income (Schippers et al., 2022), even increasing their vulnerability to viral diseases (Enichen et al., 2022).

The dysfunctional situation in most countries worldwide strengthens the incentives for mass migration into Western countries that still offer better prospects, in theory at least. However, this challenge, if mishandled, may lead to importing poverty (Martin, 2009; Murray, 2017) creating an underclass, and further proclivity of an unequal society and possibly a Death Spiral Effect (*cf.* Gomberg-Muñoz, 2012; Peters and Shin, 2022). Furthermore, there is some evidence that poverty gives rise to higher crime rates (Dong et al., 2020). In the US, even minor crimes are severely punished, and imprisonment of poor people escalates inequalities (Wacquant, 2009; Wilkinson and Pickett, 2009a).

### 2.10 Dysfunctional behavior of both elites and masses

Prior research has shown that extreme inequalities lead to dysfunctional societies, both in the animal kingdom as well as in human societies (Grusky and Ku, 2008). In the animal kingdom it has been shown to lead to “behavioral sink” or extreme dysfunctional behavior (Anderson and Bushman, 2002). In the Universe 25 behavioral experiment, mice lived in perfect conditions with enough living space, food and water, but when their numbers grew, inequalities rose and the behavior of all mice became dysfunctional and led to the extinction of the colony, long before the maximum number of mice was reached (Calhoun, 1973; Adams and Ramsden, 2011). It has been argued that in that particular experiment, where resources were plenty, the controlling of resources by a small number of mice, as well as excessive (negative) interaction led to the decline of the colony (Ramsden, 2011). Even after the numbers fell to lower than when pathology set in, mice behavior stayed dysfunctional (Ramsden and Adams, 2009). The extent to which these studies have validity for human society is certainly debatable. For obvious ethical reasons, it is for instance not possible to do a study in which a form of extreme hierarchy is tested, and subsequently lifted, but there is general agreement that countries with high inequalities have more social problems (Grusky and Ku, 2008; Van Bavel, 2016).

Historically, the elite that accelerates problematic societal developments and oftentimes is at the start of the Death Spiral Effect, either because of their greed and hunger for power, or just because power corrupts, is also getting anxious as societal decline progresses (Baker, 2022; Rushkoff, 2022). The pressure to perpetuate economic growth comes with repercussions and an inevitable crumbling of financial markets, as for instance happened in 2008 (Rushkoff, 2009). Rushkoff (2009) had hoped there would be a self-correcting mechanism when financial markets collapse, but this apparently was not the case. As the elite notice that things are going wrong, often, instead of using their wealth to make things better, they use their buffer for protecting themselves from the “masses” and for “escapism.” They start looking for ways to escape the pending societal collapse that they helped creating (Rushkoff, 2022). While the masses experience a loss of freedom and prosperity, and may desperately try to hold on to whatever property and resources they still have (desperation principle; Hobfoll et al., 2018), the elite also realizes disaster may strike and they may also get into a survival mode, and may even start to fight each other (*cf.*, Turchin, 2007).

Currently, the optimism of connectivity and the internet and the possibilities for open source democracy seem to have faded (Rushkoff, 2003). Censorship has set in, along with a loss of scientific freedom (Da Silva, 2021; Kaufmann, 2021; Shir-Raz et al., 2022). The scientific debate was stifled during the COVID-19 crisis and dissenting views were censored (Shir-Raz et al., 2022). Suppression tactics resulted in damaging careers of dissenting doctors and scientists regardless of academic or medical status (Shir-Raz et al., 2022). This has led to a loss of trust in science and institutions (Hamilton and Safford, 2021). Worse, when knowledgeable scientists with reasonable arguments and rigorous data are suppressed, this could offer ammunition to conspiracy theorists to claim that orthodox science is non-tolerant and wrong. Especially centralized censorship may increase certainty in radicalized views (Lane et al., 2021). Anyone questioning. science’ and official governmental narratives may be called a conspiracy theorist, as a way of discrediting and delegitimizing critics (Giry and Gürpınar, 2020). It has been argued that conspiracy theories are also a sign of dissatisfaction with governance, society or policies, and some conspiracy theories may turn out to be true (Swami and Furnham, 2014).

Surveillance capitalism, the collection and commodification of personal data by corporations, shifts the power of governments toward large companies (Big Other). These corporations have the power to observe and influence human thinking and decision-making for example via direct advertising. Direct advertising has become far more aggressive (Schwartz and Woloshin, 2019), especially for products of little benefit and high sales (DiStefano et al., 2023). Effective direct advertising can be guided by surveillance capitalism. This can be a tool for business (knowing customers preferences) but also an invasion of privacy (Zuboff, 2015, 2023, see also Yeung, 2018). Worryingly, strategic actors such as large corporations and governments (i.e., the elite) can unevenly influence the media (media bias), possibly leading to an increasingly narrowing of the definition of “facts/true knowledge” versus “fake news/disinformation,” e.g., stating that only governmental or other elite-endorsed narratives represent the. truth’ (*cf.* Gehlbach and Sonin, 2014). People and scientists that differ in their views from the official narrative, can be censored, marginalized and expelled, even if they are prudent in their publications and wording within the debate (*cf.* Prasad and Ioannidis, 2022). Some authors have even contended that this combination may bring us on a path toward totalitarianism (Desmet, 2022), and have called for a way to rethink and uphold democracy and democratic principles (Della Porta, 2021; Ioannidis and Schippers, 2022), as well as democratic control of technology (Gould, 1990).

A more positive solution such as a form of direct democracy is often not considered and if it is, people often feel not capable of bringing this about (*cf.* Rushkoff, 2019). In general, under such conditions, distrust escalates as the elite starts to fear the masses and the masses fear the elite and both tend to show dysfunctional behavior (*cf.* Widmann, 2022). When in phase 4 (Figure 2) resources dwindle as a result of the continuously downward spiral, the desperation principle may apply. The desperation principle has been formulated within the conservation of resources theory (COR; Hobfoll et al., 2018). In COR theory, people, organizations and societies strive to obtain and hold on to resources they value. Since resource loss is more salient than resource gain, people go to great length to prevent resource loss. However, individual and groups must invest resources in order to prevent resource loss, recover from losses and/or gain resources. When valuable resources are lost, resource gains become more important (Hobfoll et al., 2018). The desperation principle states that “When people’s resources are outstretched or exhausted, they enter a defensive mode to preserve the self which is often defensive, aggressive, and may become irrational” (Hobfoll et al., 2018, p. 106). Resource loss cycles indicate that tress and a cycle of faulty decision-making may lead to less resources to offset resources loss and these loss spirals “gain in momentum as well as magnitude.” At the same time, “resource gain spirals tend to be weak and develop slowly” (Hobfoll et al., 2018, p. 106). This indicates that once this spiral has set in, it is hard to reverse and the Death Spiral Effect may set in.

## 3 Reversing the downward spiral: upward spiral

### 3.1 Breaking free: strategies to overcome societal dysfunctional behavior

In general, grand societal challenges such as rising inequalities, social unrest and societal decline affect large portions of the population, are highly significant, but are potentially solvable (Eisenhardt et al., 2016). Lately, management scholars have applied organizational knowledge to a societal context by formulating solutions for such societal challenges using management theories (George et al., 2016), and models have been offered to integrate literature on resilience with crisis management literature (Williams et al., 2017). For instance scholars have offered solutions to alleviate poverty (e.g., Banerjee and Duflo, 2011; Mair et al., 2016) and psychological injury in the context of large conflict and wars (De Rond and Lok, 2016). With respect to decreasing inequalities, especially work by Mair et al. (2016) could be of interest, as they propose scaffolding as a way to decrease inequalities and alleviate poverty. A downward spiral may be reversed by using an adaptive response. This may be important in order to reverse the trend, but also in case of post-collapse recovery (*cf.* Bunce et al., 2009). Based on the literature cited above, the following steps may be necessarhin

Making sure that people involved are also participating in the in decision-making process is key. As Perret et al. (2020): 1 state: “The fate of states, companies and organizations are shaped by their decisions. It is then surprising that only a minority of individuals are involved in the decision-making process.” This would suggest that a rigorous change in the way societal decisions are made may be an important point to intervene (Lupia and Matsusaka, 2004; Demarest and Victor, 2022). In general, the upward spiral may start with (1) open mindedness with regards to changes necessary to break the downward spiral. These may include (2) reflection on the current and desired situation, as well as (3) the development of a strategy for the restoration of trust, rather than a focus on finding scapegoats (avoidance of a blame game can be reached for instance via a truth and reconciliation commission), and (4) involvement of more society members. This may lead to (5) higher quality decision-making and enhanced autonomy and positive ripple effects within society, and this may in turn lead to (6) decreased social inequalities and (7) a more free and open society where people can thrive and prosper. Bouncing back from adversity may then require both resilience and compassion, as described below.

### 3.2 Resilience

The concept of resilience, or how individuals, organizations and societies bounce back from adverse events, is informative (Van Der Vegt et al., 2015), and this is also mentioned as an important concept within complex adaptive systems models (e.g., Centeno et al., 2022). Resilience on all levels seem to be dependent on social integration, for instance on how supportive families and communities are, and this is especially apparent in times of crises (Banerjee and Duflo, 2011; Van Der Vegt et al., 2015). Having resilient networks is also important in this respect, and research on how to strengthen networks and communities may be key to societal resilience and rebuilding society after decline has set in (Van Der Vegt et al., 2015). Trust and compassion, as well as effective communication and collaboration within networks may enable not only more effective response to crises and disasters (Shepherd and Williams, 2014), but also reduce suffering caused by societal decline (Williams and Shepherd, 2017). After disasters, such as earthquakes, it has been found that family firms, especially those that involve more members, are best positioned to make use of posttraumatic entrepreneurial opportunities for recovery and growth (Salvato et al., 2020). Recent work in a company context has shown that companies can react to adverse events in diverse ways to post-shock challenges (Shepherd and Williams, 2022). This research highlights the role of post-adversity growth during adversity and gives insight in the different paths to resilience. This research offers insights in how to break a Death Spiral and move toward an upward spiral.

### 3.3 Compassion

As a downward spiral is often accompanied with a loss of humanness, the reversal of the downward trend will need a restauration of humanness and compassion. Compassion organizing was coined as a term to describe the coordinated organizational response to human suffering inside and outside of the organization (Dutton et al., 2006). Compassion is an innate response to human suffering, and involves recognition of suffering, empathetic concern and behavior that is aimed at alleviating suffering (Dutton et al., 2006). The reversal of a downward trend of societal decline, may be more difficult than posttraumatic growth after (natural) disasters, by its sheer scale. While a disaster may provoke compassionate organizing to alleviate mass suffering (Shepherd and Williams, 2014; Williams and Shepherd, 2017), what can be done for the alleviation of suffering and crisis management in the context of societal decline may be less obvious (*cf.* Williams et al., 2017). Often, individuals, teams and organizations working to alleviate suffering experience intense emotions that may spur strong involvement of volunteers and companies, and people often refer to this as a “calling” (De Rond and Lok, 2016; Schabram and Maitlis, 2017; Langenbusch, 2020). However, that sensemaking and strong emotion can also lead to faulty decision-making (Cornelissen et al., 2014; Hafsi and Baba, 2022). In the COVID-19 crisis, digital innovations were suggested as a way to alleviate suffering (Majchrzak and Shepherd, 2021). However, we need rigorous studies on which compassion-based interventions may be effective. It is important to help people to regain a sense of purpose in life and increase posttraumatic growth of individuals and groups in society (De Jong et al., 2020; Dekker et al., 2020).

### 3.4 Turnaround leadership and culture change

Prior research has shown that leadership is key to follower behavior (Cao et al., 2022). Passive and destructive leadership styles, such as abusive, narcissistic and authoritarian, were associated with higher levels of dysfunctional follower behavior, i.e., workplace aggression. Conversely, ethical leadership, change-oriented as well as relational-oriented leadership was negatively associated with workplace aggression. If leaders’ behavior changes, this also affects organizational culture and behavior of followers.

A historical turnaround leader that managed to get a country out of a negative spiral was Nelson Mandela, in South Africa. Instead of installing tribunals, he established the Truth and Reconciliation Commission. This helped to move beyond blame and regain respect for one another. A problem with leaders that step up in turbulent times, is that they are often not recognized and valued in the midst of the turmoil by the masses, and they may also be seen as enemies of the ruling elite. As they try to reverse the downward spiral, they may face hardship, imprisonment, and sometimes even death. As a case in point, Nelson Mandela spent over 27 years in prison.

Turnaround leadership faces the difficult task to break the negative spiral and restore trust and bring back positive energy within the organization (Bibeault, 1981) or society (Gibson, 2006). This is all the more difficult, because such companies often suffer from collective denial, or unwillingness to admit that there is a problem at all. Sometimes the problems become so big, that people act like the problem does not exist (*cf.* Meyer and Kunreuther, 2017). On a company level, it has been observed that even though individually, people know and may even admit that the company is in trouble, they collude in collective denial, or pluralistic ignorance (Kanter, 2003). Strategies that successful turnaround leaders in companies often employ are promoting dialog, engendering respect, sparking collaboration and inspiring initiative (Kanter, 2003). The challenge is how far the tactics used by a turn-around leader within an organization can be applied on a societal level as well. Without a shared vision, recovery after collapse in the context of adaptive systems is unlikely (Bunce et al., 2009).

### 3.5 Avoidance of blame game

During the COVID-19 crisis, many people started to suspect that conspiracies were at play, probably due to both the scale of events, as well as the need for explanations (Van Bavel et al., 2020; Douglas, 2021; Pummerer et al., 2021; Ivanova, 2022). While the belief in conspiracy theories has been related to reduced institutional trust, lower support for and adherence to imposed measures (Pummerer et al., 2021), it can also be seen as an ineffective form of coping with the situation (Schippers, 2020). While people may have a need for finding out who or what is to blame for the situation, the dangers of co-occurring collective narcissism (i.e., exaggerated belief in the greatness of the in-group, which is not recognized by others) and conspiracy theories, such as the endorsement of violence and undemocratic governance, have been pointed out (De Zavala et al., 2022). As the relevance and/or truthfulness of conspiracy theories are often hard to check, constructive ways forward are often blocked if these run rampant. When focusing on parties that are to blame for the situation, while some people may feel that revenge can be helpful, blame mostly fulfills a felt need for retribution and only a subset of people seems to find revenge important and even pleasurable (Szymaniak et al., 2023). Punishment of perpetrators is not very effective to prevent or retribute transgressions in terms of law enforcement (Metz, 2022). In the current situation, this may be even more complicated, as a lot of damage may have been done for the “right” reasons, i.e., in the name of public health (Schippers and Rus, 2021; Schippers et al., 2022). It may be hard to disentangle motivations of individual decision makers and decisions were also made in a context of approval of such measures (*cf.* Ohlin, 2007). A more constructive approach therefore may be in reconciliation (Metz, 2022), reversing the most aggressive and ineffective policies, and learning from mistakes in order to do better in the future (Schippers et al., 2022). However, if pressure for revenge and retribution escalates, decision-makers who made grave mistakes will likely double down on their mistakes in order to avoid punishment. As many of these decision-makers continue to have power in (or on) public health and science, such defensive continued endorsement of false narratives can be devastating for the credibility of both public health and science at large. Moreover, it is imperative that people can easily experience positive emotions instead of enduring stressors (Johnson, 2022). Preventing long-term stress is critical to quality of life and longevity (Johnson, 2022). Mutual empathy may need to be promoted in generating a positive view for the future (Beck et al., 2018; Halamová et al., 2022).

### 3.6 Civil and intelligent disobedience

Whether in families, groups, organizations or society, if people perceive that a toxic culture is ingrained or becomes apparent, the majority of people have problems addressing this, out of fear of being excluded from the group, or because they do not know how to reverse the downward trend (Packer, 2009; Richardson, 2020). Richardson (2020) describes that with a change in society toward a “new normal,” people in power will demand obedience. Concentration of power and wealth at the top is often accompanied by forcefully compelling obedience to new customs, rules, and behavior. In the early stages people often either downplay the signs of danger and may succumb to coercion, out of fear for the consequences (Richardson, 2020). People who openly resist, or criticize decisions, often face dire consequences. Effective ways of “resisting” listed by Richardson (2020) are a refusal to accept the new goals and tradition imposed, not buying into the belief that this new order is inevitable, and making a conscious choice to be rather “left behind” than to join in. This all the while maintaining civility and commitment to the common good, and adhering to values that are important to a civil society (Richardson, 2020). Constructive deviance and speaking up (as opposed to silence) are an important step in counteracting (organizational) wrongdoing (Starystach and Höly, 2021). Also, civil and *intelligent disobedience* can be ways to counteract courses of action chosen by leaders and policy makers that may hurt society or companies as a whole may hurt society or companies as a whole (Chaleff, 2015; Hughes and Burnes, 2023). Some even argue that constructive deviance and intelligent disobedience should become socially expected behavior (Ralston, 2010). This is in line with recommendations to prevent groupthink to make sure to appoint a “devil’s advocate” (Janis, 1982b; Janis, 1983; MacDougall and Baum, 1997; Akhmad et al., 2020). Interestingly, group members that strongly identify with the group are more prone to speak out on collective problems (Packer, 2009). Another action that individuals and groups can take is high-quality listening, as an antidote to polarization, which has become a huge issue in society (Itzchakov et al., 2023; Santoro and Markus, 2023). Recent research indicates that genuinely listening in order to try to understand the others’ perspective has been shown to aid depolarization, and can be. a valuable tool for bridging attitudinal and ideological divides’ (Itzchakov et al., 2023; p. 1).

### 3.7 Collective action

Besides individuals in groups and societies speaking up and voicing concerns, collective action may have additional benefits. While individual control over the social system seems out of reach, collective action can bring about positive outcomes for the group as a whole (Klandermans, 1997). Key predictors of collective action are perceived injustice, efficacy (i.e., sense of control) and identity (i.e., identification with a group; Van Zomeren et al., 2008; Van Zomeren, 2013). People are also more likely to engage in protests if they perceive injustice for the group they identify with (Klandermans, 2002). Injustice and efficacy seem to be stronger predictors for collective action in case of incidental rather than structural disadvantage, while group identification was a strong predictor for collective action for both types of groups (Kraemer, 2021). While structural disadvantages are more harmful, both psychologically and in terms of inequalities, they are less likely to evoke action-oriented emotional response and collective action (Major, 1994; Schmitt and Branscombe, 2002), and are thus harder to change (Sidanius and Pratto, 2000; Jost and Major, 2001; Sidanius et al., 2004). Such differences and structural injustices often become ingrained and disadvantaged groups may even start to see their state as natural and immutable (Major, 1994). It is then seen as a property of a certain group (Kraemer, 2021) and the existing differences between groups are seen as legitimate (Jost and Major, 2001).

Social Dominance Theory seeks to explain how and why societal group-based inequalities exist and persist, even though people would wish for a more equal society (Pratto, 1999; Pratto et al., 2006). In most societies, some groups enjoy material and symbolic resources, such as political power, wealth, and access to housing and food (Pratto et al., 2006). Both privileged as well as underprivileged groups may come to see the status quo as legitimate, and this is often institutionalized. Profit-maximizing financial institutions, internal security organizations and criminal justices systems may enhance hierarchy (Pratto et al., 2006). Conversely, human and civil rights movements and institutions, welfare organizations and religious organizations may reduce hierarchy. However, such organizations often lack funding and often do not really challenge the status quo (Pratto et al., 2006). When collective action is taken against the status quo, it is often seen as illegitimate and are shut down (Pratto et al., 2006), unfortunately repression of social movements is quite common (Loadenthal, 2016). Historically, non-violent collective actions have been more successful then violent ones in (re)instating democracy (Chenoweth and Stephan, 2008; Chenoweth, 2021), and this type of actions have become much more common (Kraemer, 2021, see also Schippers et al., 2022). Thus, breaking the downward spiral is not easy and is often jeopardized by the ruling elite.

### 3.8 Top priority: decreasing inequalities

**Figure 3:**
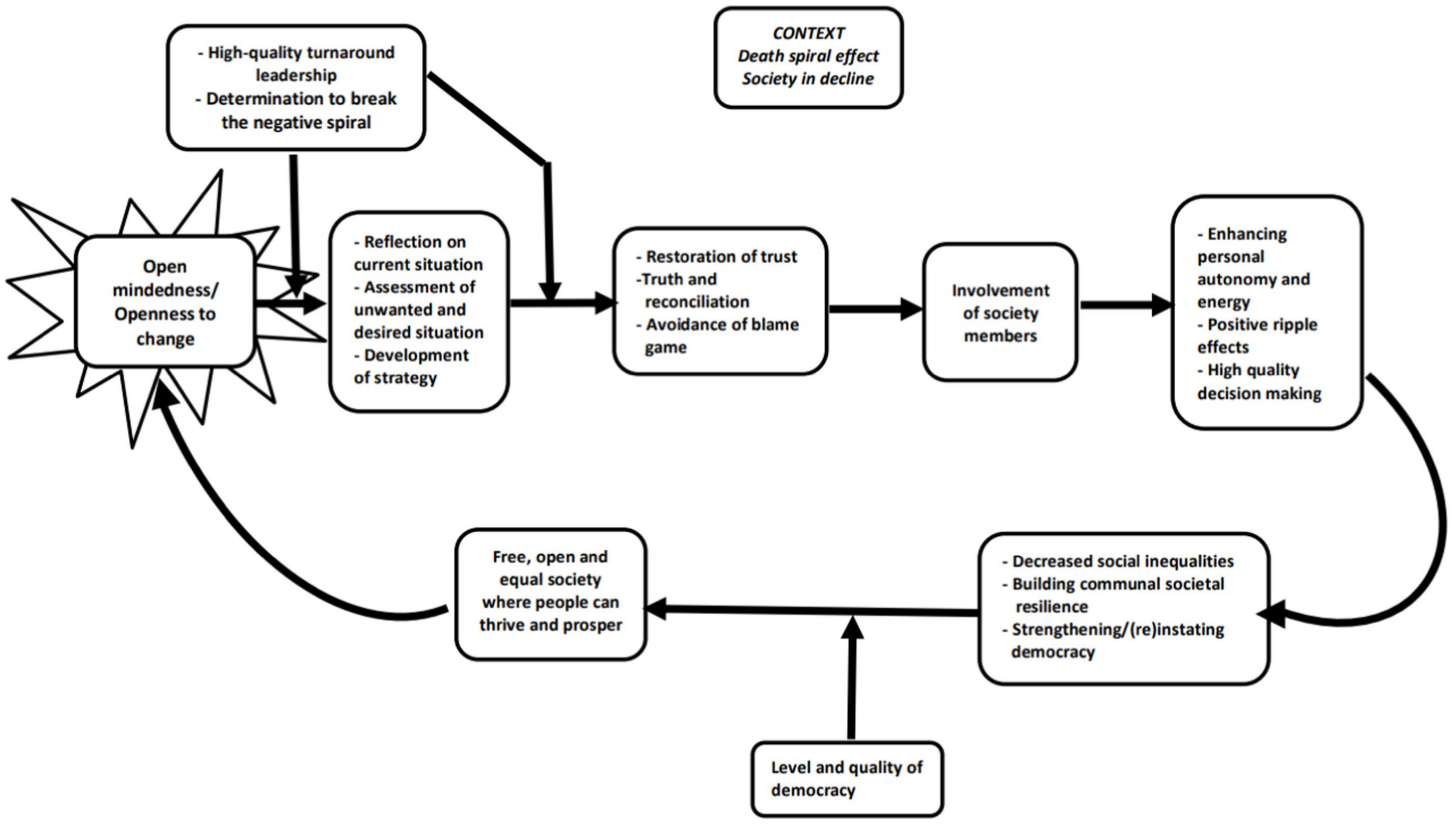
Upward spiral breaking the Death Spiral: from societal decline to societal flourishing.

As we have argued that a (large) increase in inequalities is an important marker of societal decline, this problem seems to be of utmost importance to address. In Figure 3, we depict how this can be done. In the United Nations Sustainable Development Goals, SDG10 is reducing inequalities (United Nations, 2022). However, the focus of the targets and indicators seems to be more on enhancing inclusion than on explicitly reducing inequalities (Fukuda-Parr, 2019). This is an important omission, as it would be key to address the issue of extreme inequalities and the concentration of wealth at the top (Fukuda-Parr, 2019). While it is clear from our review that rising inequalities and rising authoritarianism can contribute to significant societal decline and high levels of mortality via the Triangle of Death (disease, famine and war), it is not easy to determine where to start in order to reverse this trend. While this seems a large and complex problem, when thinking of possible solutions, it is clear that effectiveness and ease of implementation matter the most. Communities have a responsibility to investigate methods to act on the social, educational, physical, and mental health crisis. Interventions should be rigorously tested with randomized controlled trials for effectiveness and then audited for their implementation success. At the same time, complete equality should not be something to strive for, as this could kill innovation and creativity, rather, an optimal level of income differences is key (Charles-Coll, 2010). Indeed, it seems there is an optimum level of (in)equality, showing an inverted “U” shaped relationship (Charles-Coll, 2010). In situations where ordinary people are involved in shaping the response to crisis, a further widening of disparities seem to have been prevented (Van Bavel and Scheffer, 2021), and hence this seems a promising avenue in going forward.

The COVID-19 crisis and measures of unprecedented severity and duration are related to many negative side effects and increase inequalities worldwide (Marmot and Allen, 2020); hence stress, health, and trauma for vulnerable populations must be addressed (Whitehead and Torossian, 2020). It may take a long time to recover from the economic fall-out and rise in inequalities (Whitehead and Torossian, 2020). Governments should take individual and societal well-being as a spearhead for decision-making in the upcoming years (Frijters et al., 2020). Hopefully, with effective interventions, the tide can be turned and a positive spiral can be induced (e.g., Schippers and Ziegler, 2023). However, while many ideas and proposals may emerge, implementing them without rigorous trials may add further waste after we have already endorsed too many failed interventions.

## 4 Final comments

Societal demise and collapse are part of the cyclical nature of civilizations, that have historically seen a rise and fall over time. The Death Spiral Effect entails rising inequalities and rising authoritarianism, creating an elite that controls access to resources more tightly, and making decisions that may set humanity on a path to famine, war and disease. If the trend of decline is not reversed, the aftermath of a possible collapse will become evident, and society may be unable to achieve post-collapse recovery.

In short, our review, synthesizing research from several fields, indicates that next to turnaround leadership and building resilient communities, using compassion, avoiding a blame culture and strengthening of democracy may help. Ideally, governments, companies, all relevant stakeholders as well as individuals should collaborate toward the goals of a healthier and happier future for all.

## Author contributions

MS played the primary role in the conception of the manuscript, writing, reviewing, and revising the manuscript. JI contributed to writing the manuscript and editing the manuscript. ML contributed to and partly wrote the section on “Differences from other concepts,” crafted Table 1, contributed to writing, and editing the manuscript. All authors contributed to the article and approved the submitted version.

## Footnotes

^1^Turchin spells it asabiya.
